# Exam‐level analysis of lecture capture viewing and student exam performance

**DOI:** 10.1002/2211-5463.70133

**Published:** 2025-09-30

**Authors:** Kirk Hillsley

**Affiliations:** ^1^ Biology Department Trent University Peterborough Canada

**Keywords:** biology, education, lecture capture, lecture recording, lecture videos

## Abstract

Lecture capture (LC) systems offer students flexible review of lecture content, but their impact on learning outcomes remains mixed. LC engagement and exam performance were analyzed in three in‐person courses with LC videos posted for review, each with three lecture blocks and three independent noncumulative exams. Zoom analytics and exam grade data were collected for 299 students across 982 noncumulative exam observations. Four LC metrics were derived per exam: total view duration, number of lectures viewed, number of unique views, and days between access and exam. Average exam scores were compared between LC viewers (*n* = 216) and nonviewers (*n* = 83): LC viewers scored significantly higher than nonviewers (66.1% vs. 59.4%). A linear mixed‐effects model with student‐level random intercepts showed opposing effects of total viewing time (+1.74% per hour) and number of lectures viewed (−1.92% per lecture), implying that average LC view duration per lecture (total minutes watched ÷ lectures viewed) was the strongest predictor of exam score. A *post hoc* median split of average LC view duration per lecture indicated an 8.02% higher score for students above the median. Decomposition of total LC view time revealed a between‐student effect on exam grade (+2.52% per hour) and a within‐student effect (−0.84% per hour), showing that spikes above a student's own average view time are associated with a lower exam grade. These findings align with self‐regulated learning theory, demonstrating that while greater LC viewing time generally benefits performance, its impact depends on strategic, habitual engagement rather than episodic cramming.

## Graphical abstract

This study asks how lecture capture relates to exam performance in biology courses with three noncumulative exams. Mixed‐effects modeling separated between‐students from within‐student effects on exam %. Students who watched more recordings earned higher grades, but viewing spikes above a student's own mean, consistent with cramming, predicted small declines in exam %.

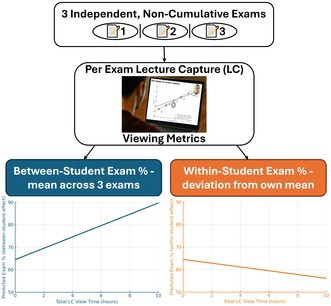

**Abbreviations**
CI: confidence interval
LC: lecture capture
LMS: learning management system
OLS: ordinary least squares
REML: restricted maximum likelihood
SE: standard error
SRL: self‐regulated learning

In contemporary higher education, the integration of technology into traditional pedagogical frameworks has become increasingly prevalent, with lecture capture (LC) systems emerging as a ubiquitous tool aimed at enhancing the learning experience [1]. LC technologies systematically record live lectures, capturing both audio and video, and providing students with resources for on‐demand review [2]. The rationale behind implementing LC is multifaceted, encompassing the accommodation of diverse learning styles, the provision of flexible learning opportunities, and the facilitation of deeper engagement with course material beyond the confines of the classroom [3]. By furnishing students with comprehensive archives of lecture content, these systems transcend the limitations of traditional note‐taking and in‐class comprehension, fostering a more dynamic and personalized learning environment that empowers students to actively construct their knowledge at their own pace and according to their individual needs. While the adoption of LC has been driven by the expectation of improved academic outcomes, the empirical evidence regarding its actual impact on student performance remains mixed, thus requiring a nuanced examination of the factors that mediate the relationship between LC engagement and academic achievement [4].

The adoption of LC technologies aligns with the principles of universal instructional design by providing broader access to educational resources and increasing flexibility for students to manage their learning experiences [5]. This inclusivity in educational delivery can benefit students with diverse needs, including those requiring accommodations due to health‐related issues. Indeed, LC provides significant public health benefits by enabling students to access educational content remotely when they are unwell, thus reducing the spread of illnesses on campus [6]. This additional value of LC technology has become much more apparent since the COVID‐19 pandemic [7].

Students generally have a positive perception of LC due to its flexibility, convenience, and its perceived role in improving learning and academic performance [8, 9]. Students find lecture recordings to be useful as a study and review tool, especially if they have to miss classes [10]. This flexibility can benefit students with disabilities, reducing anxiety and creating a more inclusive learning environment [11]. Moreover, LC enables students to revisit lectures, aiding comprehension and knowledge retention, which can be particularly advantageous for students facing challenges in traditional classroom settings [12]. Additionally, LC can support students who require additional time to process information or who benefit from repeated exposure to complex concepts, promoting fairness in educational outcomes [13]. As such, LC can also be viewed through the lens of equity and justice for students.

Although the majority of students access recorded lectures when available, their engagement is typically selective. For example, fewer than two‐thirds of lectures were accessed in large health courses [14], and in a large geology course it was reported that viewers skipped around mid‐video rather than watching end‐to‐end [15]. Recent data found clusters of different student viewing behaviors in educational technology students, but with the majority of video views being brief at 7 min [16].

This selective use is further concentrated around exams, not initial lecture delivery. Multiple investigations document sharp spikes in viewing immediately before assessments: over half of all LC accessions occur within 24 h of an exam, while same‐day review of new material is rare [14, 15, 17, 18, 19, 20]. This last‐minute, high‐volume viewing may reflect surface‐level cramming associated with poorer outcomes. Finally, viewers of LC are far from a monolithic group. A notable minority of top performers choose never to view recordings, relying instead on live attendance, peer collaboration, or traditional text‐based study [14, 21]. Together, these engagement patterns underscore that while LC fulfills vital flexibility and review functions, actual student behavior spans a spectrum from deep, targeted use to minimal or nonuse, a diversity that any evaluation of LC's impact must accommodate.

A growing body of observational research has not reached consensus on whether providing LC recordings improves student exam performance. Some studies have shown LC to be correlated with a decrease in student performance [4, 22, 23], particularly when students use recordings as a replacement for attending lectures [24, 25]. Other studies show no significant effects on exam scores or overall course grades with LC [26, 27, 28]. Subgroup analyses within these studies often identify benefits for specific groups of students. For instance, those from non‐English speaking backgrounds may find recorded lectures particularly useful [14], as might students with learning disabilities who might require more time to absorb information [21]. Finally, some studies show LC improves student performance [29, 30, 31], particularly in students who use recorded lectures as a supplement to attending live lectures rather than as a replacement [32].

The lack of consensus on the question of whether LC videos benefit student performance may reflect the fact that many of these studies are reliant on self‐reported data or student surveys, which are likely to be inaccurate due to sampling/selection bias [30]. However, in the relatively few studies using video access data logs to determine the relationship between LC viewing behavior and exam performance, there remains a lack of consistent findings. Zhang *et al*. [33] reported that the usage of recordings is positively related to academic performance in finance students. Similarly, a study on economics students found a 4% improvement in exam performance with LC video usage [18]. However, in bioscience students, there was no relationship between LC usage and examination performance [34]. This mixed perspective suggests that the effectiveness of LC varies significantly, likely depending primarily on student behavior.

Self‐regulated learning (SRL) theory emphasizes students' active planning, monitoring, and adaptation of their study strategies [35, 36], skills that are especially important for academic success and the effective use of LC tools [37]. LC recordings offer a powerful tool for SRL by enabling learners to revisit material on their own schedule and selectively review challenging segments [38]. However, for effective SRL, students must plan when and how to engage with LC videos in ways that align with principles of distributed practice and metacognitive monitoring [39, 40]. For example, habitual, evenly spaced viewing may reflect deliberate planning and self‐monitoring, whereas last‐minute ‘cramming’ spikes may indicate surface‐level engagement with limited strategic regulation.

The present study tests SRL‐informed hypotheses about how different LC recording usage is associated with exam performance. In each of the courses studied, there were three blocks of lectures, each with an independent noncumulative exam. This allowed the study of LC engagement at the individual‐exam level and partitioning between‐student (habitual) vs. within‐student (spike) viewing patterns. Through this SRL lens, it was hypothesized that differential LC viewing would be associated with differential exam performance. Specifically, it was predicted that there would be (i) a positive between‐student association, where students who consistently integrate LC into their study routines outperform peers, and (ii) a negative within‐student association, where abrupt increases in viewing, indicative of cramming, relate to lower exam scores.

## Methods

This study involved the secondary use of fully anonymized educational data and was exempt from Research Ethics Board review, in accordance with Article 2.4 of the Tri‐Council Policy Statement: Ethical Conduct for Research Involving Humans [41].

This study was undertaken in three undergraduate biology courses taught by the same instructor. Data from two third year courses and one first year course were used for this study, with the first year course being for nonbiology majors. Each course had 10 weekly 2‐hour in‐person lectures throughout the semester, with two midterms and a final exam. Students were informed at the beginning of each course that testing would prioritize understanding lecture content rather than recall. All exams were closed book, taken in proctored settings, primarily composed of multiple‐choice questions testing understanding and some recall, with a minority of short‐answer items testing higher cognitive levels. Each of the exams was noncumulative, so for any given lecture, the lecture material was only tested in a single exam. Each in‐person lecture was also recorded on Zoom and posted on Blackboard LMS within 24 h.

At the end of the semester after all grades were released, data analysis was started. Final course grades were downloaded from the Blackboard LMS. LC usage was downloaded from the Zoom Recording Analytics interface, and all data were merged into Excel files. Each individual LC usage by a student recorded the date of access and the duration of the viewing time. These data were supplemented in Excel with the date of when the lecture occurred and when the lecture material was tested. For each student exam observation, we computed two timing variables from the raw Zoom logs: the days from a student's first LC access to the lecture date (to index early review) and the days from the last LC access to the exam date (to index cramming). Because these timing measures were discrete and non‐normal, all timing‐exam associations were tested with Spearman's rank correlation, reported as *ρ*(df) = value, *P* = value.

Four primary LC viewing metrics were derived for each student exam observation (*n* = 982 total exam rows from 299 students across three courses). Each metric was calculated as the total for LC videos of all lectures being tested for any given exam. (1) Total View Duration—sum of all minutes spent watching LC videos for a given exam. (2) Lectures Viewed—number of distinct lectures accessed with a duration of ≥ 1 min for a given exam. (3) Unique Views—total count of unique LC video accessions for a given exam. (4) Days Before Exam—the elapsed days between the LC access and the relevant exam date, with a value of zero equating to an LC video access on the day of the exam.

At the student level (*n* = 299), each student's average exam percentage was calculated across their three noncumulative exams, and LC viewing metrics were aggregated across all three exams. These semester‐aggregated descriptive statistics were calculated for all students and for viewers and nonviewers. Students who never accessed any LC videos in each course (across all three exams) were classified as nonviewers; all others were viewers. Statistical significance was assessed at *α* = 0.05 throughout. All data shown are mean ± standard error of the mean (SEM).

### Statistical analysis

Bivariate associations between LC metrics and exam % were examined via Spearman's *ρ* and/or ordinary least‐squares regression (with 95% confidence bands from statsmodels in Python). Pearson correlations and VIFs in Python were used to quantify high collinearity among the four LC predictors. A *post hoc* sensitivity analysis in G*Power 3.1 indicated that, with 299 students (982 exam observations), the study had 80% power (*α* = 0.05) to detect small effects of *f* ^2^ = 0.02, equivalent to noticing a change in exam % of roughly 2 points per additional hour of LC viewing.

The primary multivariate analyses used linear mixed‐effects models [restricted maximum likelihood (REML)] implemented in Python's statsmodels MixedLM. The model included a random intercept for each student to account for within‐student correlation across their three exams. All fixed‐effect predictors remained in their original (uncentered) scale; the model was estimated via REML. This same model was repeated for LC viewers only, too. The model formula was:

$${\text{ExamPct}}_{\mathrm{ij}}=\begin{array}{l} \beta 0+\beta 1\;{\text{TotalViewDuration}}_{\mathrm{ij}}\\ +\;\beta 2\;{\text{LecturesViewed}}_{\mathrm{ij}}+\beta 3\;{\text{UniqueViews}}_{\mathrm{ij}}\\ +\;\beta 4\;{\text{DaysBeforeExam}}_{\mathrm{ij}}+\left(1|{\text{Student}}_{i}\right) \end{array}$$

where ExamPct_*ij*_ is the percentage score of student *i* on their *j*th exam, followed by that same student's viewing metrics for that exam + the random intercept for student *i* (1∣Student_*i*_). In both full‐sample and decomposition models (below), the random intercept and residual error were assumed to be normally distributed with variances *σ* ^2^_student_ and *σ* ^2^_resid_, respectively. Model assumptions were evaluated by inspecting residual *Q*–*Q* plots and Shapiro–Wilk tests for normality (all *P* > 0.05), Breusch–Pagan tests for homoscedasticity (*P* > 0.10).

All predictors and outcomes were computed at the exam level: each row represents a single exam for a given student (Exam 1, Exam 2, and Final), and all LC metrics (minutes, lectures viewed, unique views, and timing) refer to the lectures relevant to that exam only. As exams were noncumulative and sampled nonoverlapping lecture material, the primary model pooled exam blocks. As a sensitivity analysis, exam‐block fixed effects (categorical indicators for Exam 1/Exam 2/Final) were added to the model. Results of these checks did not alter the substantive conclusions; pooled estimates are therefore reported in the text.

In addition to these mixed‐effects models, the total LC view duration was decomposed into two uncorrelated components to separate stable between‐student differences from exam‐to‐exam within‐student fluctuations. For each student, we calculated their mean total view time across the three exams and then computed, for each exam, the deviation of that exam's view time from the student's own mean. These two variables, the student‐level mean and the exam‐level deviation, were entered as fixed effects in a linear mixed‐effects model (with a random intercept for student), estimated by REML with the following formula:

$${\text{ExamPct}}_{\mathrm{ij}}=\begin{array}{l} \gamma 0+\gamma 1\;¯T¯V¯Di+\gamma 2\left(\mathrm{TVD}\mathrm{ij}-¯T¯V¯Di\right)\\ +\;\left(1|{\text{Student}}_{i}\right) \end{array}$$

where ¯T¯V¯Di is student *i*'s mean total view duration across their three exams, and (TVD*ij*−¯T¯V¯Di) is the within‐student deviation for exam *j*.

To visually demonstrate the distinct within‐ and between‐student effects uncovered by our mixed‐effects analysis, a small synthetic dataset closely matched to our real estimates from all students was generated. Five ‘students’ were assigned baseline LC view times spanning the middle of the observed range. For each student, three exam observations were simulated: two clustered tightly around their personal mean viewing time and a third displaced by +1 h. Exam scores were calculated using the model's estimated intercepts and slopes—so that the between‐student trendline intercepts the *y*‐axis at approximately 59.9% and the aggregate trendline at approximately 64.7%—and then perturbed by Gaussian noise with a standard deviation equal to the residual SD from the full mixed‐effects model. This procedure produced 15 points which faithfully represent the actual data.

For the *post hoc* analysis, among all exam rows in which a student watched at least one lecture for that exam (*n* = 511), the average LC view time per lecture was calculated. These LC viewers were median split, and Welch's two‐sample *t*‐test was used to test for statistical significance. This same subset of LC viewers was also divided into three equal‐sized tertiles, and a one‐way ANOVA was calculated. No formal adjustment was made for multiple comparisons across these distinct analyses, as each served a different, exploratory, *post hoc* purpose.

All statistical codes are available upon request. Mixed‐effects models were fitted using statsmodels' MixedLM module with REML estimation. *T*‐tests and ANOVAs were performed via SciPy's ttest_ind (Welch's option) and *f*_oneway functions, respectively.

## Results

### Descriptive statistics

Data are reported from 299 unique students who collectively sat 982 individual exams and accessed LC videos 2545 times in three different courses. Of the 299 students, 216 (72.2%) opened at least one LC recording over the three exam blocks in each course (‘viewers’), whereas 83 (27.8%) never accessed a single LC video (‘nonviewers’). The average LC video posted was 108 ± 0.9 min in duration. The LC viewing metrics and exam % for these student populations are summarized in Table 1. LC viewers on average watched 45% of the total length of LC videos available and accessed 60% of the individual lectures available.

**Table 1:** Student‐level lecture capture engagement and exam performance. Each row summarizes a single student's aggregate performance and recorded‐lecture behavior across three noncumulative exams in one or more courses. ‘Nonviewers’ (*n* = 83) never accessed any lecture captures; ‘Viewers’ (*n* = 216) accessed at least one recording across the term. Days Before Exam’ is computed only for exam rows with ≥ 1 LC access; nonviewers are NA and do not contribute. ‘Viewers' reports the student‐level mean (average across viewer students). ‘All’ reports the event‐weighted mean across all access events (pooling all viewings across all students and exams).

	All	Nonviewers	Viewers
*n*	299	83	216
Exam grade	64.3 ± 0.7%	59.4 ± 1.2%	66.1 ± 0.8%
View duration (% of total)	352.7 ± 23.8 min (32.5%)	—	488.3 ± 27.8 min (45%)
# Lectures Viewed (*% of total*)	4.3 ± 0.2 (43%)	—	6.0 ± 0.3 (60%)
# Unique Views	8.3 ± 0.6	—	11.5 ± 0.7
# Days Before Exam	2.4 days	—	3.3 days

The majority of LC viewing behavior was as a study tool for exams. Of the 2545 total individual LC video accesses, 54.5% of views occurred on the day of, or the day before the exam. By contrast, only 2.9% of LC views occurred on the day of, or the day after the lecture. There was a consistent pattern of students accessing LC recordings of lectures in a sequential order prior to each exam, with the earliest lectures being tested in each exam accessed 5.8 ± 0.3 days before the exam, compared with 1.6 ± 0.1 days for the last lecture (*P* < 0.001, unpaired *t*‐test).

**Fig. 1:**
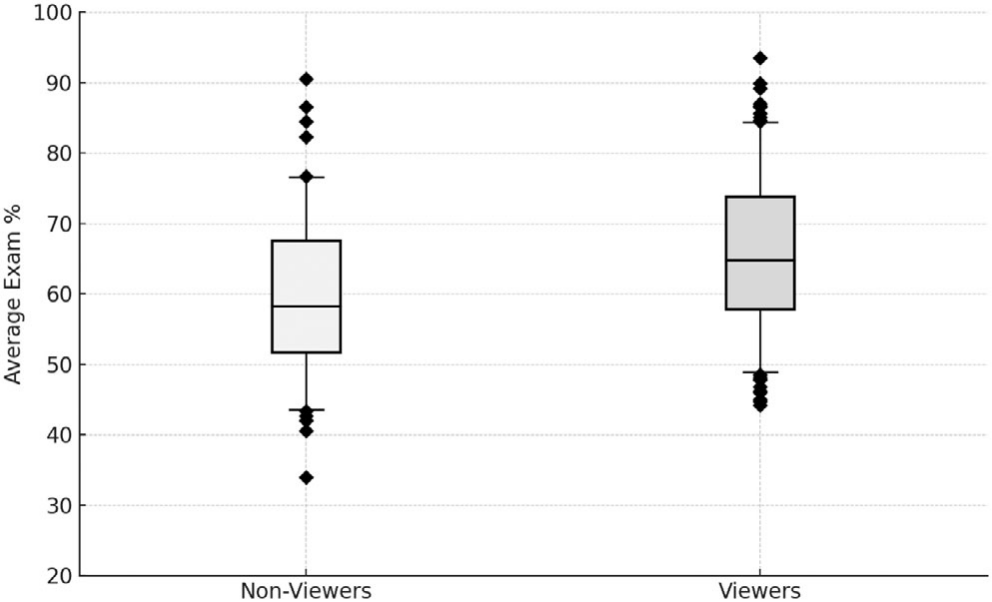
Average exam % by lecture capture (LC) viewer status (*n* = 299). Boxplots compare each student's mean exam percentage (averaged over three exams) by whether they ever accessed any lecture capture during that course/semester. Each box edge marks the 25th/75th percentiles, whiskers at 5th/95th percentiles, black diamond outliers, solid black median lines.

**Fig. 2:**
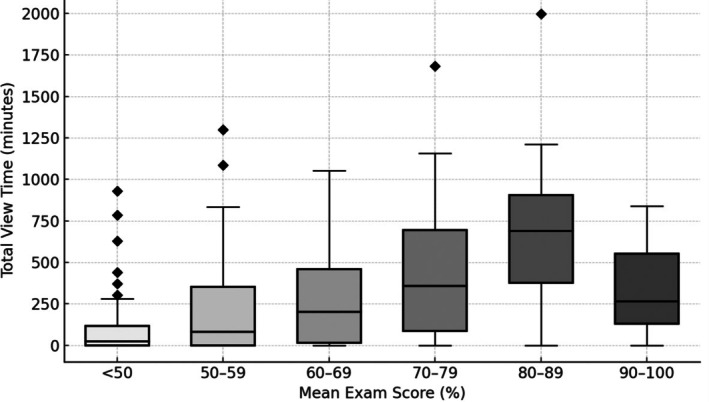
Total view time by mean exam score bins. Boxplots show the distribution of total lecture capture viewing time (in minutes) for students grouped by their mean exam percentage: < 50%, 50–59%, 60–69%, 70–79%, 80–89%, and 90–100%. The central line in each box denotes the median; the top and bottom of the box indicate the 75th and 25th percentiles, respectively; whiskers extend to the most extreme values within 1.5× the interquartile range; and diamonds represent outliers (data points beyond the whiskers). Analysis is based on *N* = 299 student–course observations.

Aggregate data from all 299 students showed (Fig. 1) that LC viewers averaged higher exam performance (66.1 ± 0.8%) than nonviewers (59.4 ± 1.2%), which suggests an association between LC viewing and student grades. To further test the consistency of this association, the disaggregated data of exam‐level observations were used to determine whether viewing behavior predicts performance in the full dataset with each student taking 3 independent exams per course. Correlation confirmed a positive association between total minutes watched and exam percentage *β* = +0.018% per minute, Spearman's *ρ* = 0.26 (df = 980, *P* < 0.001), corresponding to an aggregate slope of +1.74% per hour. To further illustrate this association, the mean exam grades of all students per course (*n* = 299) were binned as shown in Fig. 2. There is a general increase in total view time (over the semester) as exam score increases up to 89%. However, this trend reverses in the highest scoring students (90+%), mainly due to a significant minority (38%) of these students never accessing LC videos.

Taken together, these student‐level and exam‐level data demonstrate a general positive association between viewing LC recordings and student exam performance. However, LC viewing metrics had high multicollinearity: Pearson r among Total View Duration, Lectures Viewed, and Unique Views ranged from 0.75 to 0.81 (all *P* < 0.001), with Days Before Exam uncorrelated (|*r*| < 0.07). Corresponding VIFs were 6.14, 5.83, 6.69, and 2.21, respectively, confirming that the three duration/count metrics share substantial variance. Hence, a mixed‐effects modeling approach was used on the full 982 exam observations to estimate each metric's partial effect while accounting for within‐student and between‐student variability.

### Mixed‐effects model results—All students

A linear mixed‐effects model (REML on raw data) was fit to the 982 exam‐level observations, with a random intercept for each student (*n* = 299 unique IDs) and fixed effects for four LC viewing metrics. Table 2 displays the full set of parameter estimates.

**Table 2:** Mixed‐effects model estimates for all students (*n* = 982 exams). Linear mixed‐effects model with random intercepts for Student ID and fixed effects for Total View Duration (minutes), Lectures Viewed (count), Unique Views (count), and Days Before Exam. CI, 95% confidence interval; *P*, two‐tailed *P*‐value; SE, standard error; *t*, *t*‐statistic; *β*, estimated partial effect on Exam %.

Predictor	*β* (coef.)	SE	95% CI	*t*	*P*
Intercept	64.51	0.82	[62.90, 66.12]	78.61	**< 0.001**
Total View Duration (mins)	0.029	0.008	[0.013, 0.045]	3.63	**< 0.001**
# Lectures Viewed	−1.92	0.74	[−3.37, −0.47]	−2.59	**0.010**
# Unique Views	−0.58	0.34	[−1.25, 0.09]	−1.71	0.087
# Days Before Exam	0.028	0.025	[−0.02, 0.08]	1.12	0.262

Values in bold are statistically significant (*p* < 0.05).

From the mixed‐effects model, the student‐level random‐intercept variance was estimated at *σ* ^2^_student = 82.1, and the residual variance at *σ* ^2^_residual = 149.12, yielding an ICC of approximately 0.355. This indicates that 35.5% of the total variability in exam percentages was attributable to stable, between‐student differences (e.g., overall ability or unmeasured traits), while the remaining 64.5% reflects within‐student fluctuations from one exam to the next and other unmodeled factors.

**Fig. 3:**
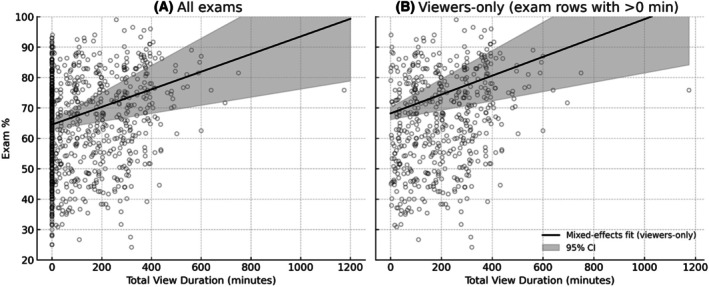
Total view duration vs exam %: all vs. viewers only. Panel A shows all exam‐level observations (*n* = 982). Panel B restricts to exam rows with > 0 min of viewing (viewers only; *n* ~511). Solid line = mixed‐effects prediction; shaded band = 95% CI. Removing nonviewers reduces the large spike at 0 min and yields a slightly higher intercept and similar positive slope, consistent with Table 3.

**Fig. 4:**
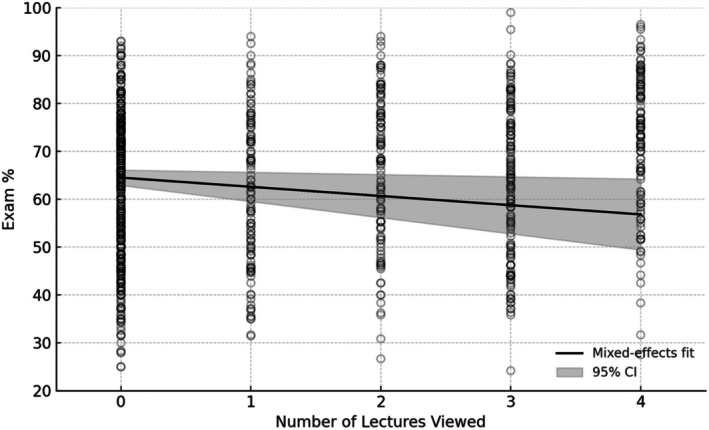
Lectures viewed (per exam) vs. exam %. Scatterplot of all 982 exam‐level observations (each point is one exam for one student). The solid black line shows the adjusted partial effect from the mixed‐effects model (holding total minutes, unique views, and timing constant), which is negative. By contrast, the unadjusted (bivariate) association between lectures viewed and exam % is positive in the raw data; it is not plotted here to avoid overplotting but is reported in the text. The shaded gray ribbon indicates the 95% CI around the model‐based prediction. *X*‐axis values are per‐exam counts of distinct lectures viewed.

In this mixed‐effects model, total LC view duration emerged as a strongly positive predictor (*β* = 0.029) as represented in Fig. 3, indicating that each additional hour of recorded‐lecture watching is associated with a 1.74% increase in exam score, holding all other factors constant. In addition, there was a statistically significant negative partial effect (*β* = −1.92) predicting that when other variables are held constant, viewing one additional LC recording is associated with an estimated 1.92% decrease in exam score, as shown in Fig. 4. Although the unadjusted scatter shows a positive trend, the line plotted in Fig. 4 is the model‐based partial effect for lectures viewed and is therefore negative when other viewing metrics are held constant, consistent with Table 2.

The number of unique LC views and the number of days before exam did not reach significance in the mixed‐effects model. Thus, repeated plays of the same material and earliness of LC access did not independently predict exam scores once total minutes and lecture count were accounted for. Adding course fixed effects (first‐ vs. third‐year) left the key coefficients qualitatively unchanged (direction and significance), indicating the pattern holds across course years.

While this model confirms a clear positive link between LC time and performance, its single slope may mask two opposing phenomena. So the total LC view duration was next decomposed into student‐level means and exam‐level deviations.

### Between‐ vs. within‐student effects

**Fig. 5:**
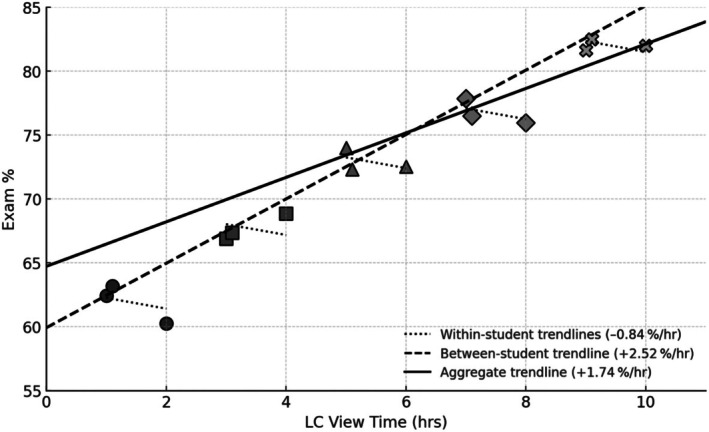
Scatterplot of hypothetical exam scores vs. lecture capture (LC) view time for five students (A–E), each with three independent exams. Symbols denote individual exam outcomes for each student (two clustered lower LC‐view times and one isolated higher LC‐view time). Short dotted lines show within‐student linear fits (slope = −0.84% per hr), illustrating that deviations above a student's own average viewing predict slightly lower performance on average. The black dashed line is the between‐student trend (slope = +2.52% per hr; intercept ~ 59.9% at 0 h), indicating that students with higher average viewing tend to score better overall. The black solid line is the aggregate (raw) trend (slope = +1.74% per hr; intercept ~ 64.7% at 0 h), which combines both within‐ and between‐student effects.

To disentangle the aggregate positive effect of LC view time, the mixed‐effects model was refitted (see Methods) with two predictors: each student's mean LC view time (between‐student component) and the deviation of each exam's view time from that student's mean (within‐student component). This produced a strong positive between‐student effect (*β* = +2.52%/hr, *P* < 0.001) alongside a negative within‐student effect (*β* = −0.84%/hr, *P* = 0.002). For comparison, the raw aggregate model remained positive (*β* = +1.74%/hr, *P* < 0.001). To summarily illustrate this contrasting data, a small, 15‐point synthetic dataset of five ‘students’ each taking three exams was generated, which mirrors the key parameters of the real dataset and has the three trendlines overlaid. This synthetic dataset is shown in Fig. 5.

### Mixed‐effects model results—LC viewers only

This same mixed‐effects model was repeated on only LC viewers to check whether the model was skewed by the nonviewer data. Table 3 displays the full set of parameter estimates. The pattern of associations is consistent with, but somewhat stronger than, the full‐sample results: each additional minute of total viewing above a student's average remains positively associated with exam performance (*β* = +0.031 ± 0.008, *P* < 0.001), and each extra lecture viewed above average continues to predict a lower score (*β* = −2.87 ± 0.71, *P* < 0.001). Similarly, unique play counts and days before exam do not reach statistical significance once minutes and lecture counts are held constant.

**Table 3:** Mixed‐effects model estimates for lecture capture (LC) viewers (*n* = 719 exams). Linear mixed‐effects model with random intercepts for Student ID and fixed effects for Total View Duration (minutes), Lectures Viewed (count), Unique Views (count), and Days Before Exam. CI, 95% confidence interval; *P*, two‐tailed *P*‐value; SE, standard error; *t*, *t*‐statistic; *β*, estimated partial effect on Exam %.

Predictor	*β* (coef.)	SE	95% CI	*t*	*P*
Intercept	68.20	1.02	[66.20, 70.19]	67.03	**< 0.001**
Total View Duration (mins)	0.031	0.008	[0.016, 0.046]	4.11	**< 0.001**
# Lectures Viewed	−2.87	0.71	[−4.27, −1.47]	−4.02	**< 0.001**
# Unique Views	−0.50	0.32	[−1.13, 0.11]	−1.58	0.113
# Days Before Exam	0.029	0.025	[−0.02, 0.08]	1.14	0.253

Values in bold are statistically significant (*p* < 0.05).

### *Post hoc* analysis—LC viewers only

**Fig. 6:**
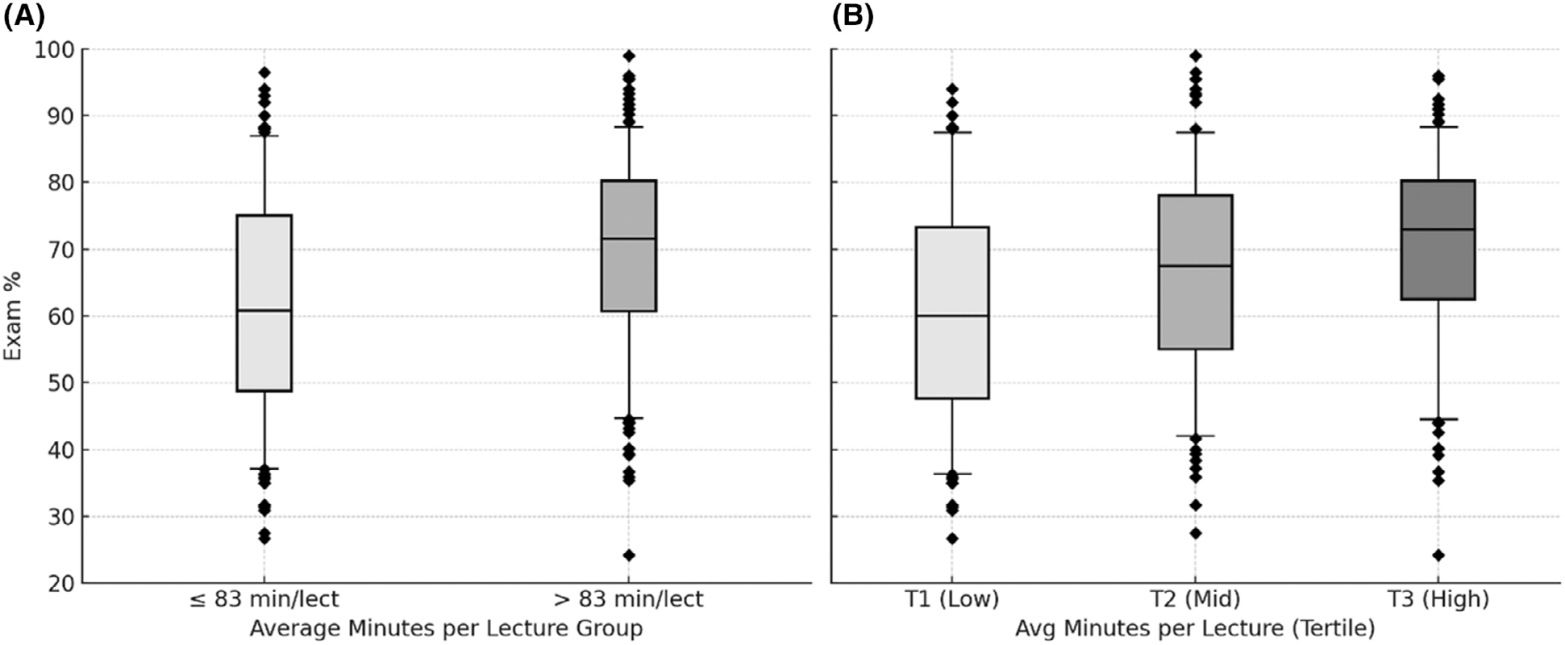
Exam percentage by average minutes of lecture capture (LC) viewing per lecture. Boxplots summarize exam‐level observations in which a student viewed ≥ 1 lecture for that exam (*n* = 511; nonviewer exam rows are excluded by definition). The per‐exam metric plotted on the *x*‐axis is Average Minutes per Lecture (AvgMinPerLecture = TotalViewDuration ÷ LecturesViewed, in minutes). (A) Observations are split at the dataset median of 83 min per lecture (≤ 83 vs. > 83 min/lecture). (B) The same viewer‐only exam data are grouped into equal‐sized tertiles of AvgMinPerLecture (T1 = low, T2 = mid, T3 = high). All boxes show the 25th–75th percentiles; the solid line is the median; whiskers span the 5th/95th percentiles; diamonds mark outliers beyond the whiskers.

As total view duration and number of lectures viewed have significant but opposing effects in the model, we performed a *post hoc* analysis using a combined metric, the average minutes per lecture (AvgMinPerLecture = TotalViewDuration / LecturesViewed). The aim of this analysis was to capture and illustrate the main findings of this study in a single metric. It represents data from LC viewers only (as number of lectures viewed is required to be > 0). This *post hoc* analysis is illustrated in Fig. 6, with Fig. 6A showing a median split of average minutes per lecture, and Fig. 6B showing the same data binned into tertiles. A Welch's *t*‐test showed the median split gave a significant difference in exam % (*t*(510.7) = −6.051, *P* < 0.0001), with those above the median LC viewing minutes per lecture scoring 8.02% higher than those at or below the median (69.74 ± 0.87% vs. 61.72 ± 1.00%, respectively). The same data binned into tertiles (Fig. 6B) show there is a progressive increase in exam % with increase LC view time per lecture. A one‐way ANOVA comparing exam percentages among the three tertiles showed a significant difference between each of the three groups (*F*(2, 508) = 16.3, *P* < 0.0001), with exam % rising from 60.90 ± 1.25% in the lowest tertile to 70.22 ± 1.06% in the highest. This linear trend across tertiles (slope ~0.0435% per minute) approximates to predict a 2.6% increase in exam grade for every hour of LC view time, consistent with the between‐student effect.

## Discussion

There are three consistent trends in how students used LC videos in this study. First, students overwhelmingly take advantage of LC recordings when they are provided. Across disciplines, roughly 70–80% of undergraduates access posted videos [13, 15, 42, 43], mirroring the current finding that 72% of students accessed at least one LC video in a course. This broad uptake suggests that, for many learners, especially those juggling course conflicts or part‐time work, LC recordings offer essential flexibility [8, 44].

Second, most students do not watch the entire LC video, as has previously been reported [45, 46]. In our sample, ‘viewers’ accessed on average only 45% of total available video minutes and accessed 60% of all lectures. This is not problematic if students are using LC recordings as a supplemental resource, but may be problematic if students are using LC videos in place of attending lectures [32, 47]. From an SRL standpoint, such selective engagement often reflects deliberate strategic planning, a core phase of self‐regulated learning, where effective learners set specific goals (e.g., reviewing only challenging sections) and direct attention purposefully [36, 48].

The third trend concerns when LC viewers access videos in relation to exams. Most students are watching LC videos as an exam study tool, with the mean viewing time being 3 days before the exam, and 54% of all LC video views occurring on the day of, or the day before the exam. In contrast, a mere 2.9% of LC views occurred within 24 h of the live lecture. This is consistent with other studies showing peaks of LC usage prior to exams [15, 17, 18, 46, 49]. SRL theory distinguishes optimal distributed practice (spacing study sessions over days) from massed practice (last‐minute cramming), so it is clear that most students are using LC videos in a suboptimal manner. In the present study, students started to study the first lecture to be tested in an exam block 6 days before the exam, and the final lecture 1 day before the exam. This upward shift in study timing aligns with SRL's self‐evaluation and strategic adaptation phases, where learners reflect on prior performance, identify knowledge gaps, and adjust their review behaviors over time, behaviors linked to stronger academic outcomes [35, 50].

The data show LC viewers averaged 6.2% more (66.1% vs. 59.9%) on exams than nonviewers, in the same range as prior positive effect sizes of 4–8%, including a study using video viewing logs [17]. Although ‘viewers’ tend to outperform ‘nonviewers’ by ~6% in this study, top performers often eschew recordings altogether. In this study, 38% of students averaging ≥  90% on exams were students who never viewed any LC videos. From informal classroom observations (attendance was not formally tracked), these high‐performing students were regular in‐person lecture attendees throughout the semester. This general pattern has also been reported in engineering [14] and medical contexts [15]. Their success underscores the heterogeneity of study approaches; recorded lectures are a valuable supplement for many, but not a universal necessity, highlighting the value of situating LC research within a theoretical framework, such as SRL to account for individual differences and inconsistent findings [51].

The REML mixed‐effects model used in this study allows for partitioning of student‐level random‐intercept variance from residual exam‐to‐exam variation and clarifies how different viewing metrics simultaneously influence performance. This analysis approach was necessary as the different LC viewing metrics displayed high multicollinearity, and each student took multiple exams. In this model, the timing of LC views did not reach significance once minutes and lecture count were included. However, it should be noted that the distribution of LC access was sharply clustered on the day of or day before the exam. This restricts the timing variable's effective range and likely attenuates its partial effect. Moreover, any potential cramming‐related variance would most likely be absorbed by total view time. Total LC view duration emerged as a robust positive predictor of exam performance: each additional hour of LC viewing corresponded to a 1.74% increase in exam score (*P* < 0.001). Similar data were seen using a mixed‐effects model on LC viewers only (1.86% exam grade increase per hour of LC viewing), highlighting that the model was not significantly skewed by the ‘nonviewer’ category.

The positive association between LC viewing time and exam % was not seen in a similar study using video access logs and a mixed‐effects model [34]. Brackenbury [34] found in a cohort of UK bioscience students no association between LC viewing and exam performance. In both the current study and Brackenbury's [34], the incoming GPA of students and attendance data were not available for study, so differences in the student populations and student behaviors may underlie these differing results.

Although total LC view time was associated with higher exam scores in this study, once total minutes were held constant, each additional distinct lecture viewed predicted a significant decrease of 1.92% in performance. These same effects were also observed in the LC viewer‐only subset. Simply put, a student who spent 2 h viewing one lecture on average scored higher on exams than a student who split those same 2 h across multiple lectures. SRL theory emphasizes that expert learners not only decide when to study, but how to study [35, 36]. Allocating 2 h to one lecture may reflect elaboration and organization strategies, actively making sense of a single topic, whereas spreading those same 2 h across many lectures can lapse into superficial skimming. The *post hoc* analysis on the combined metric of ‘average minutes per lecture’ confirmed these results, with those in the highest tertile scoring nearly 10% higher on exams than those in the lowest tertile. This simply illustrates our mixed‐effects model's core finding, the ‘minutes vs. lectures’ trade‐off, in a more tangible way rather than prescribing LC viewing times. These findings suggest that in the context of the current study, a focused, in‐depth review of specific lectures was more effective than a superficial overview of numerous lectures.

However, studies on different student populations in different learning environments have reported the exact opposite results. Owston *et al*. [14] showed that students who fast‐forwarded to sections of the videos and watched them once achieved significantly higher results than those who watched the entire recording, suggesting that in their context the higher achievers used the videos only to clarify specific topics. This aligns with SRL adaptive strategies where different tactics are used depending on their current needs. Other qualitative investigations have revealed nuanced motivational factors driving students' engagement with LC videos, categorizing them into strategic learners who leverage recordings for clarification and passive learners who may substitute attendance with video consumption [4]. Such motivational dimensions remain challenging to capture quantitatively but are essential for a more holistic understanding of LC utility [28].

The contrasting within‐ and between‐student effects on exam performance reveal a nuanced story about how students use recorded lectures. On the one hand, students who habitually devote more time to reviewing recordings outperform their peers: each additional average hour of viewing is associated with a 2.5% gain in exam score. On the other hand, when an individual student spikes their viewing above their own norm, a pattern consistent with last‐minute ‘cramming’, their score dips by roughly 0.8% per extra hour of LC viewing. This contrasting pattern aligns with SRL theory, which predicts that deliberate, habitual study behaviors bolster performance, whereas reactive, massed ‘cramming’ undermines deeper learning [35, 36].

A review of the academic literature indicates that this may be the first study to directly allow for discernment of between‐ and within‐student effects. In aggregate, these two opposing forces combine into a modestly positive raw association (+1.7% per hour), masking the fact that steady engagement and episodic over‐viewing have very different relationships to learning outcomes. Consistent with Voelkel *et al*. [52], students commonly use LC both to catch up on missed content and for pre‐exam study. This decomposition helps reconcile these uses: students who habitually review more perform better overall (between‐student effect), yet within a student, last‐minute additions to their usual viewing predict slightly lower scores (within‐student effect).

Overall, the combined results of this study showed that more LC viewing is associated with positive student exam performance; however, splitting the same study time between more individual lectures, or spikes in an individual student's LC view time, negatively affects exam %. These nuanced results suggest that for optimal student outcomes, when LC videos are made available in courses, this should be accompanied by guidance on their use. To transform LC into a true metacognitive support, instructors could embed SRL scaffolds, such as prompts to set explicit viewing goals, recommended pacing schedules, and self‐check quizzes tied to video segments, so that students actively plan, monitor, and evaluate their review rather than default to surface‐level cramming.

It is important to note that observational studies like these cannot prove causality, since motivated high performers may simply be more likely to watch LC videos. A limitation of this study is that there was no access to data on incoming GPA, one of the biggest variables that likely underlies a lot of the variability in the data. It should be noted though, that some studies have reported that access to LC videos is associated with higher exam scores, even after controlling for previous exam performance (for example [2]). Class attendance is another variable with a major influence on student outcomes [53]. Although anecdotally it was clear that lecture attendance was low during this study, formal attendance logs were not taken, so the potential influence of LC, exam %, and attendance can only be speculated upon. It is possible that the negative effect of spikes in LC viewing by individual students could reflect a decrease in lecture attendance, but these spikes could also reflect trouble understanding lecture material, for example. There are other potential confounding factors too, such as peer‐study activities or textbook use. Ideally, future studies would be able to capture all of these variables in addition to using video access logs and exam % in a mixed‐effects model.

While LC access logs do not fully capture student engagement, they offer the most practical and nonintrusive proxy for studying LC learning behaviors at scale. Zoom analytics do not capture which portions of a video are viewed, the playback speed videos are viewed at, or give any information on video engagement. This restricts insights into students' specific usage and engagement patterns and is a further limitation of this study. This type of data would be extremely valuable for both research interpretation and instructional feedback.

One further limitation of this study is the issue of generalizability. First, all of the courses in this study were biology courses. However, it is of note that one of the courses was an introductory biology course for nonbiology majors, and that the other courses contain psychology, nursing, kinesiology, and forensic science majors in addition to biology majors too. Although there are some possible consistency advantages to the courses in this study all being taught by the same instructor at the same institution, this is a clear limitation. While effects seen in this study align with other disciplines, replication in different fields and instructional settings is needed. Nevertheless, the convergence of 4–8% advantages in multiple contexts indicates that LC recordings can serve as an effective additional learning tool.

## Conclusion

This quantitative study suggests that lecture recordings can be a valuable tool for enhancing student exam performance. While increased viewing time was generally associated with higher grades among viewers, some top‐performing students did not access LC recordings at all. Therefore, rather than viewing recorded lectures as essential, educators might consider their provision through a lens of equity and justice, ensuring accessibility for all students. In line with SRL theory, educators could further optimize equity by embedding supports that help students plan, monitor, and evaluate their use of LC videos. Further research is needed to fully understand the complex and multifaceted impact of LC engagement on student learning outcomes.

## Conflict of interest

The author declares no conflict of interest.

## Author contributions

KH conceived of this research project, undertook all data analysis and interpretation, and wrote the manuscript.

## Data Availability

Data are available at https://doi.org/10.5061/dryad.vmcvdnd59.
